# Supplementary figures and images for: Lifelong dietary protein restriction accelerates skeletal muscle loss and reduces muscle fibre size by impairing proteostasis and mitochondrial homeostasis
Source: Redox Biol. 2023 Dec 2;69:102980. doi: 10.1016/j.redox.2023.102980 (PMC10755587; doi:10.1016/j.redox.2023.102980)

## Slide 1
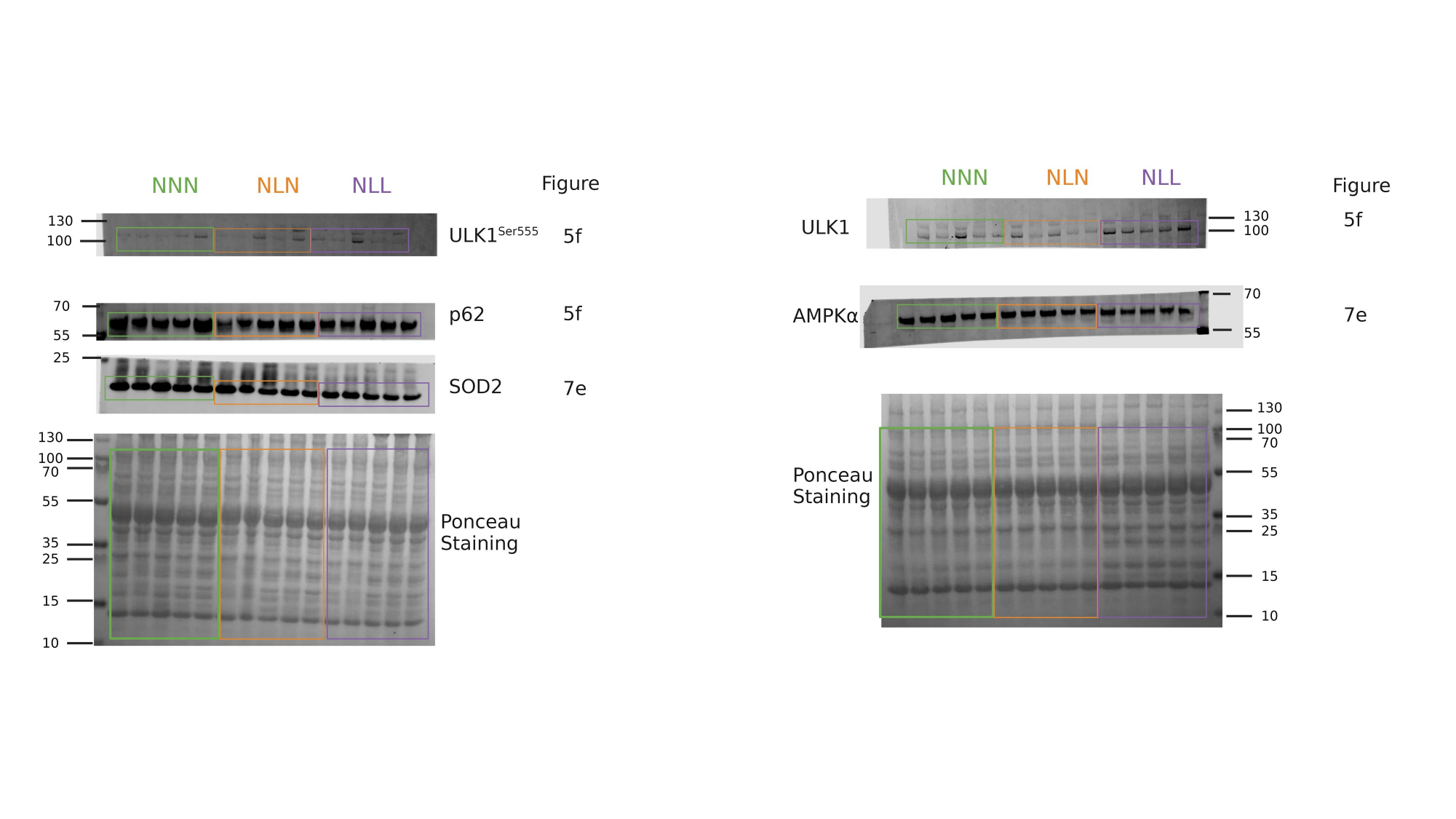

## Slide 2
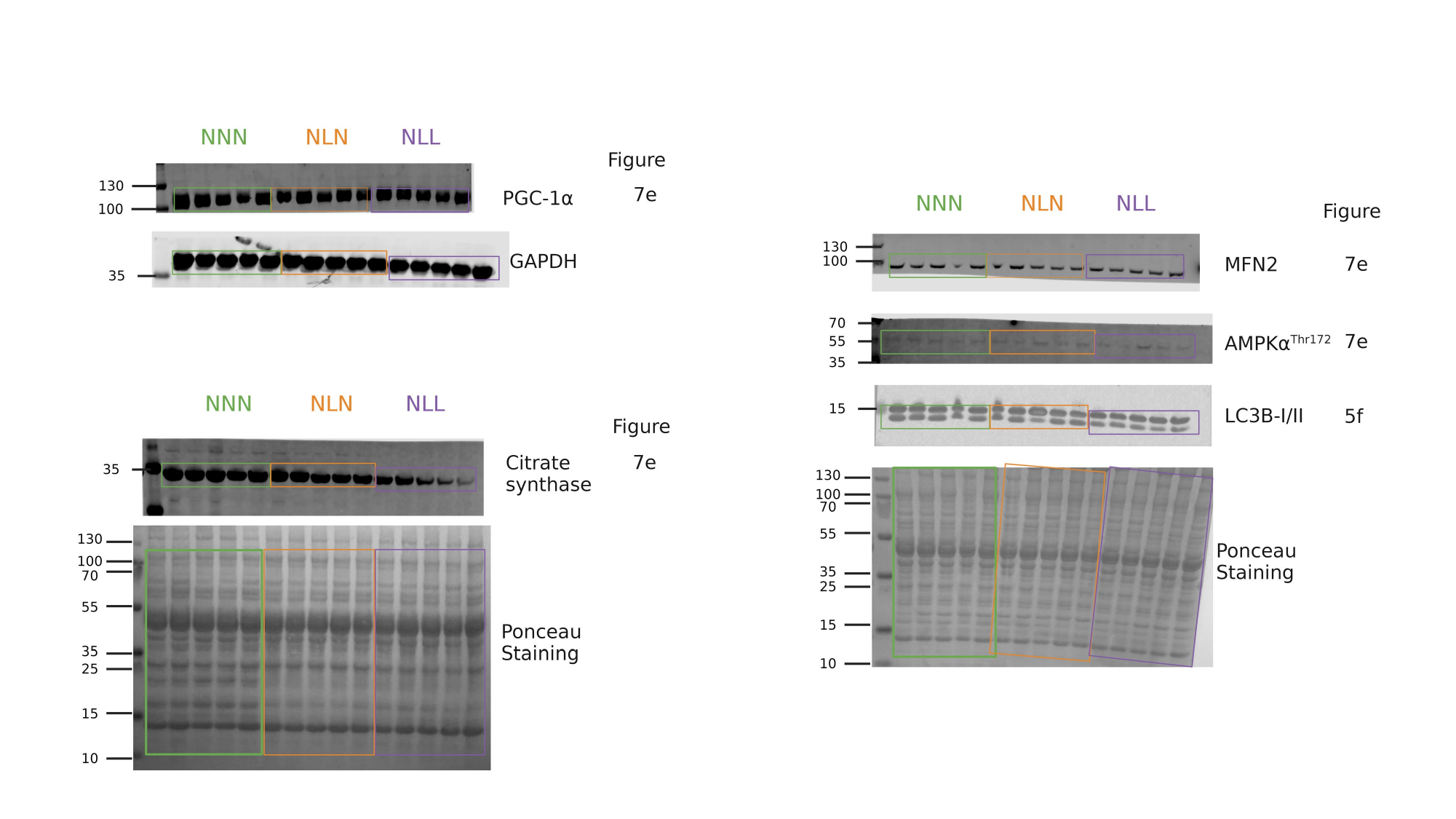

## Slide 3
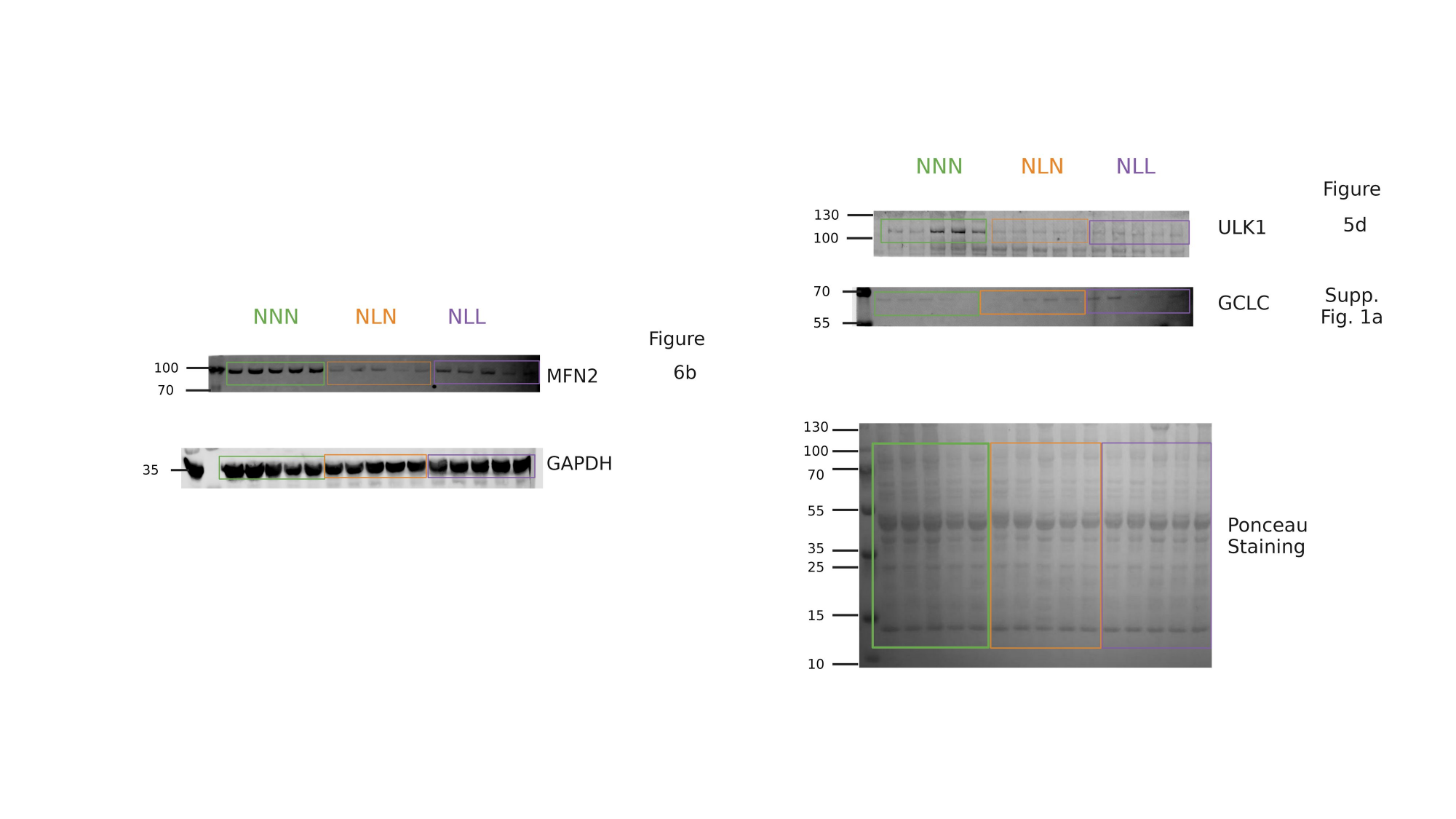

## Slide 4
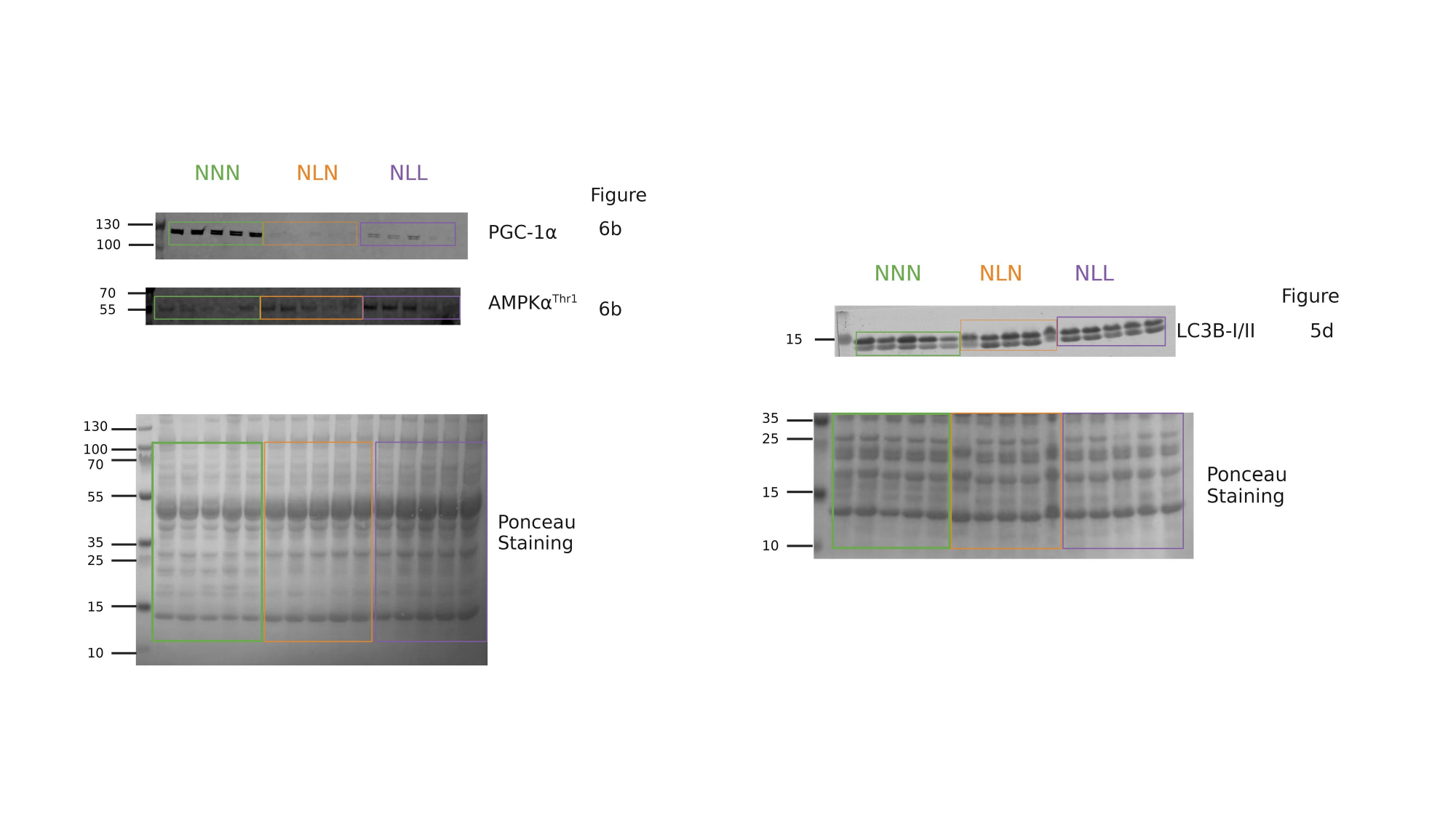

## Slide 5
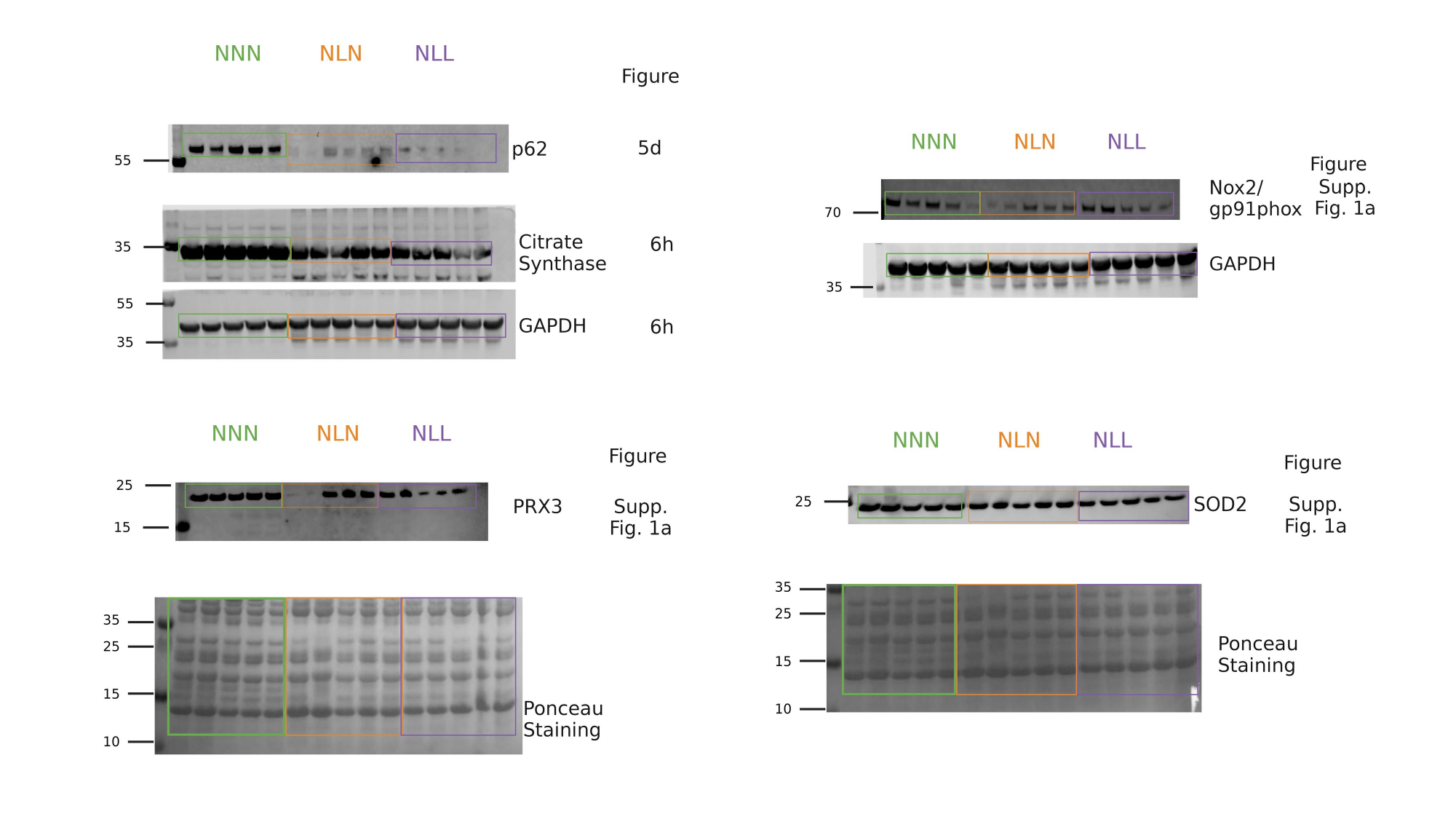

## Slide 6
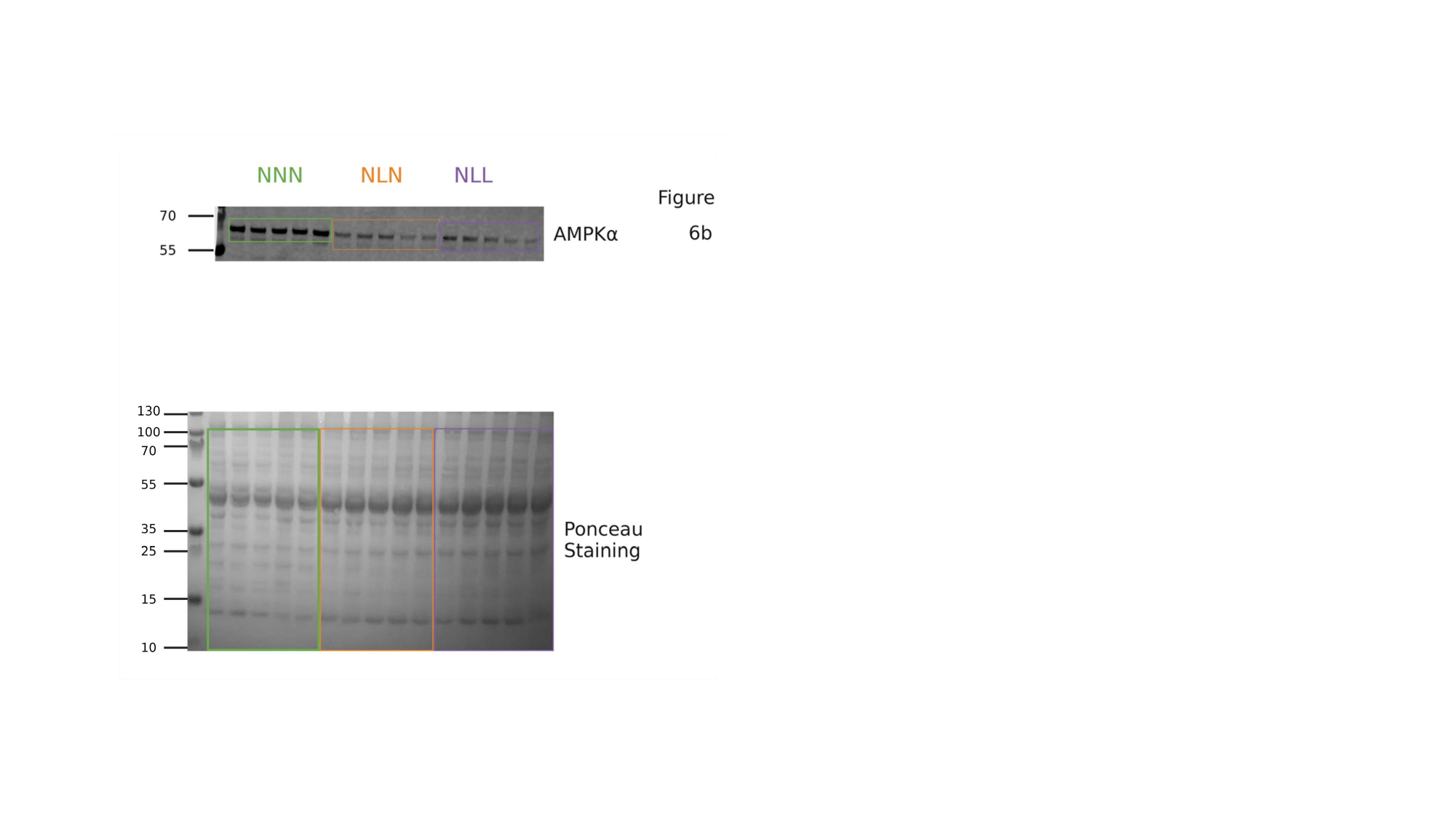

Supplement: Multimedia component 1 [file mmc1.pptx]
